# Gene expression profile of human T cells following a single stimulation of peripheral blood mononuclear cells with anti-CD3 antibodies

**DOI:** 10.1186/s12864-019-5967-8

**Published:** 2019-07-19

**Authors:** Isabel Garcia Sousa, Kelly Cristina Rodrigues Simi, Manuela Maragno do Almo, Maryani Andressa Gomes Bezerra, Gero Doose, Tainá Raiol, Peter F. Stadler, Steve Hoffmann, Andréa Queiroz Maranhão, Marcelo Macedo Brigido

**Affiliations:** 1 Department of Cell Biology, Institute of Biological Sciences, University of Brasilia, Brasilia, Brazil; 2 Instituto de Investigação em Imunologia, Instituto Nacional de Ciências e Tecnologia (iii-INCT), Brasilia, Brazil; 3 Molecular Biology Graduation Program, Institute of Biological Sciences, University of Brasilia, Brasilia, Brazil; 4 Molecular Pathology Graduation Program, Medicine Faculty, University of Brasilia, Brasilia, Brazil; 5 Bioinformatics Group, Department of Computer Science and Interdisciplinary Center of Bioinformatics, University of Leipzig, Leipzig, Germany; 6 Interdisciplinary Center for Bioinformatics, University of Leipzig, Leipzig, Germany; 7 Fiocruz Brasilia, Oswaldo Cruz Foundation (GEREB/Fiocruz), Brasilia, Brazil; 8 Max-Planck-Institute for Mathematics in the Sciences, Leipzig, Germany; 9 Santa Fe Institute, Santa Fe, NM USA; 10 Computational Biology Group, Leibniz Institute on Ageing - Fritz Lipmann Institute (FLI) and Friedrich-Schiller-University Jena, Jena, Germany

**Keywords:** Anti-CD3, RNA-seq, Antibody therapy, Regulatory T cells, Antibody engineering

## Abstract

**Background:**

Anti-CD3 immunotherapy was initially approved for clinical use for renal transplantation rejection prevention. Subsequently, new generations of anti-CD3 antibodies have entered clinical trials for a broader spectrum of therapeutic applications, including cancer and autoimmune diseases. Despite their extensive use, little is known about the exact mechanism of these molecules, except that they are able to activate T cells, inducing an overall immunoregulatory and tolerogenic behavior. To better understand the effects of anti-CD3 antibodies on human T cells, PBMCs were stimulated, and then, we performed RNA-seq assays of enriched T cells to assess changes in their gene expression profiles. In this study, three different anti-CD3 antibodies were used for the stimulation: two recombinant antibody fragments, namely, a humanized and a chimeric FvFc molecule, and the prototype mouse mAb OKT3.

**Results:**

Gene Ontology categories and individual immunoregulatory markers were compared, suggesting a similarity in modulated gene sets, mainly those for immunoregulatory and inflammatory terms. Upregulation of interleukin receptors, such as IL2RA, IL1R, IL12RB2, IL18R1, IL21R and IL23R, and of inhibitory molecules, such as FOXP3, CTLA4, TNFRSF18, LAG3 and PDCD1, were also observed, suggesting an inhibitory and exhausted phenotype.

**Conclusions:**

We used a deep transcriptome sequencing method for comparing three anti-CD3 antibodies in terms of Gene Ontology enrichment and immunological marker expression. The present data showed that both recombinant antibodies induced a compatible expression profile, suggesting that they might be candidates for a closer evaluation with respect to their therapeutic value. Moreover, the proposed methodology is amenable to be more generally applied for molecular comparison of cell receptor dependent antibody therapy.

**Electronic supplementary material:**

The online version of this article (10.1186/s12864-019-5967-8) contains supplementary material, which is available to authorized users.

## Background

Immunosuppressive therapies based on monoclonal antibodies (mAbs) started in the 1980s, with the use of Muromonab-CD3 (OKT3), an antihuman CD3 antibody, for attaining long-term graft survival after organ transplantation [1]. After decades of use, this biopharmaceutical was withdrawn from clinics due to its toxic side effects [2]. However, the emergence of a new generation of (re)engineered recombinant antibodies has sparked hopes that anti-CD3 antibodies may again be used to induce peripheral tolerance [3], renewing the enthusiasm for CD3-targeted therapies. Hence, anti-CD3 therapy is now being tested for several autoimmune and inflammatory diseases [4, 5]. Furthermore, recent clinical data on the use of Teplizumab in type I diabetes [6] contribute to this optimism that new anti-CD3 therapies for autoimmunity and transplantation will become available in a foreseeable time. The administration of anti-CD3 antibodies induces the general activation of T cells, which may lead to a state of tolerance not yet fully understood [3, 7]. The proposed mechanism of a peripheral tolerance induction rests upon a potential modulation of regulatory lineages of the CD4 phenotype [8, 9], even though CD8 regulatory cells were also shown to be affected [10, 11]. Peripherally induced regulatory cells control the activation of T cells, promoting negative feedback in the inflammatory response. The induction of a more regulatory environment by anti-CD3 antibodies could produce antigen-specific tolerance and alleviate the immune response. More recent data on human clinical data suggest that other mechanisms such as T cell exhaustion [11, 12] or the induction of inhibitory receptors on T cells [13, 14] could also contribute to the suppression of the immune response.

The effect of anti-CD3 therapy has been addressed in different studies trying to elucidate its mechanism by assessing the genetic profile of T cells induced by those antibodies. These studies have been performed by microarray analysis [15-17] or, more recently, by Next Generation Sequencing (NGS) [18, 19]. Nevertheless, in the majority of the investigations, anti-CD3 is not the unique stimulus but is combined with anti-CD28 antibody and/or interleukins, such as IL2. More importantly, these studies are often performed using isolated T cells and thus are in a very different context from the PBMC environment. In the present work, we compared two recombinant antibody fragments, a chimeric fragment and a humanized fragment in an FvFc format, with their prototypic antibody OKT3. The treatment was performed using healthy human donor PBMCs in vitro. The global changes in the transcriptome profile were assessed using RNA-seq. Subsequently, their T cell differentiation markers and immunoregulatory signatures were compared. Our data showed that, despite the antibody format, the three anti-CD3 antibodies induced a common pattern of gene expression strongly enriching regulatory genes as well as genes involved in inhibitory signaling. We propose that these comparative analyses could be exploited as a validation tool in designing new and more effective CD3-binding molecules.

## Results

### Global change in the gene expression profile in human T cells induced by anti-CD3 treatments

To compare the effects of each of the three anti-CD3 antibodies on human T cells, the gene expression profiles were analyzed. T cells were obtained from 72-h untreated or treated PBMCs with one of the three anti-CD3 antibodies: OKT3, FvFc M (OKT3 scFv fused to human IgG1 Fc), and a humanized version of this FvFc (FvFc R). Anti-CD3 was used as the sole stimulus. To avoid any further stimulation, T cells were obtained by negative selection, using magnetic beads for cell surface markers. The purity of the T cell population was assessed by flow cytometry and was above 96% (Additional file 1: Figure S1). The transcriptomes of stimulated and unstimulated T cells from a single individual were obtained by performing sequencing in two replicates. More than 55 million paired-end reads of 150 bp length were obtained. The reads were mapped to the human reference genome (hg19); of the total reads, 84 to 94% were mapped (Table 1).

**Table 1:** Read mapping rates and statistics for RNA-seq data

Replicate	All Reads	Mapped Reads	% Mapped Reads
Unstimulated (1)	59 623 760	54 990 450	92
Unstimulated (2)	61 444 968	58 149 032	94
FvFc R (1)	57 712 398	54 065 899	93
FvFc R (2)	54 633 094	51 012 088	93
FvFc M (1)	62 274 364	58 117 856	93
FvFc M (2)	65 142 278	60 452 698	92
OKT3 (1)	60 724 236	51 446 623	84
OKT3 (2)	59 435 924	50 336 034	84

**Fig. 1:**
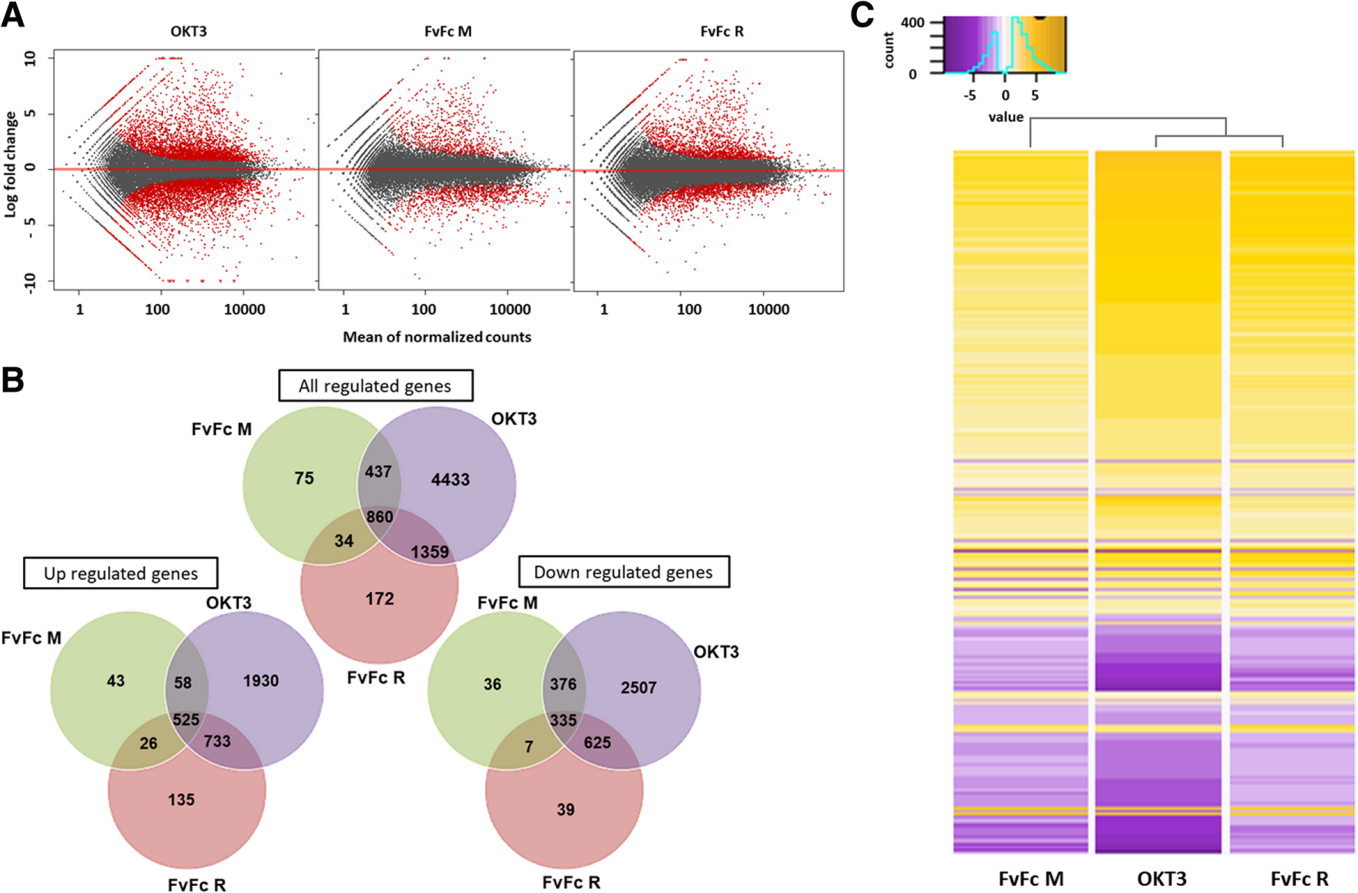
Global gene expression profiles in anti-CD3-treated human T cells by RNA-seq data. The transcriptome was obtained in CD3 T cells collected from a healthy donor at two different moments, treated or untreated with anti-CD3 antibodies. Only genes with padj ≤ 0.05 were considered differentially expressed. **a** MA-plot with the global gene expression profile; red dots indicate up- or downregulated genes. **b** Venn diagrams showing an overlap of regulated expressed genes compared to the control, among different anti-CD3 treatments. **c** Clustering analysis and heatmap of gene expression based on fold change data. Cluster analysis was performed with 860 commonly regulated genes (shown in rows) for each sample (columns). Gradient colors from purple to gold represent lower to higher expression (range from − 9.27 to 9.22)

Subsequently, we assessed differentially expressed genes (DEGs) by comparing each anti-CD3 antibody-treated sample with the control of unstimulated T cells. The gene sets found to be differentially expressed in the different treatments are shown as a MA plot in Fig. 1a and as a Venn diagram in Fig. 1b. OKT3 treatment resulted in a larger set of differentially expressed genes (7089) with a fold change of less than − 0.8 or above 0.8, followed by FvFc R treatment with 2425 DEG and FvFc M treatment with 1406 DEG. We found 860 genes that were equally regulated among the treatments, considering a padj ≤ 0.05. Except for FvFc R treatment, DEGs were mostly downregulated. FvFc R induced the most unbalanced DEG dataset, with 58% (1,419) upregulated over 41% (1,006) downregulated DEGs. The gene regulation profile promoted by FvFc R was more similar to OKT3 than FvFc M, even though the cluster analysis suggested a similar DEG profile for each treatment (Fig. 1c).

### Associations of DEGs with gene ontology categories

**Fig. 2:**
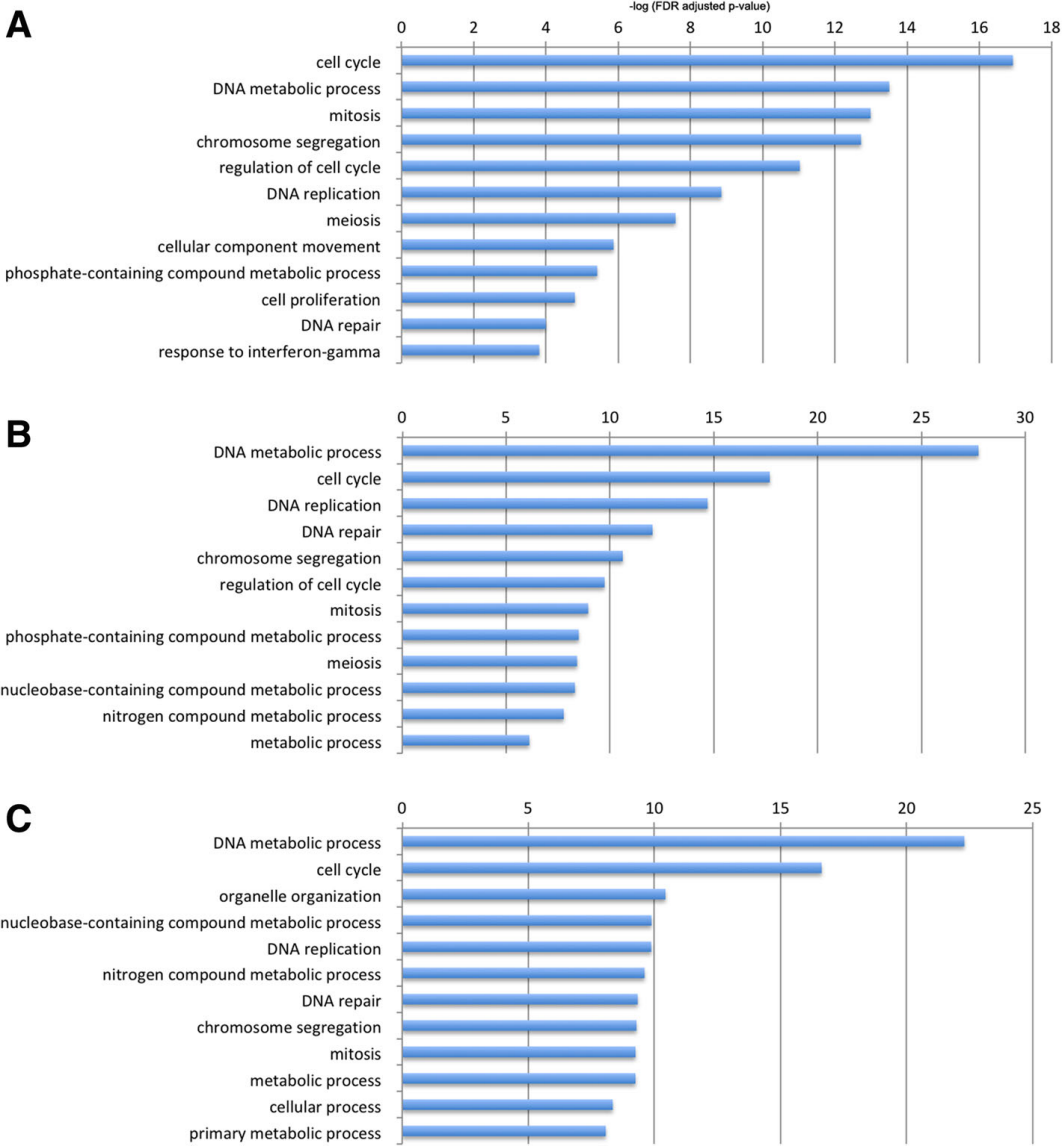
Gene set enrichment analysis of differentially expressed genes. Gene ontologies associated with upregulated genes in peripheral blood CD3 cells following anti-CD3 treatment. The top twelve enriched biological process categories were calculated using Panther. GO terms associated with cell proliferation was found to be overrepresented. **a** FvFc M-treated, (**b**) FvFc R-treated, and (**c**) OKT3-treated T cells

**Fig. 3:**
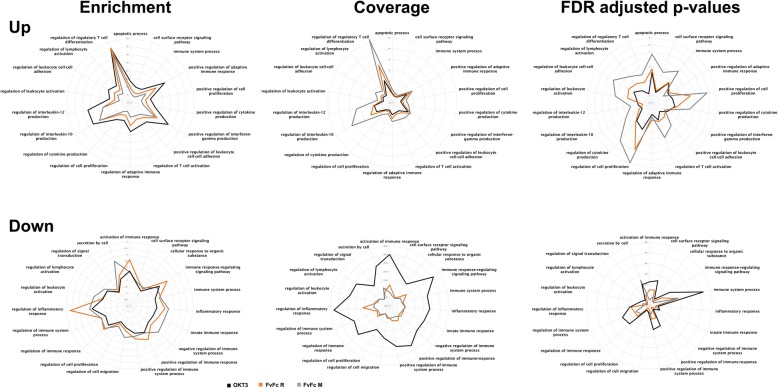
Gene set enrichment analysis of differentially expressed genes associated with immune terms. Radar Plot of the GO term profile enrichment, coverage (completeness) and FDR adjusted *p*-value of immune-associated terms. The terms were selected among up- and downregulated DEGs for each antibody treatment, accessing those associated with immune response and inflammation typically associated with anti-CD3 therapy. The black line represents OKT3 treatment; the orange line, FvFc R treatment; and the gray line, FvFc M treatment

Anti-CD3 stimulation was shown to affect different set of genes [18, 19]. Therefore, functional characterization of the differentially expressed genes was performed using GO term enrichment analysis. Anti-CD3 activated and repressed DEGs were separately classified for the GO category “biological process”. Upregulated genes were dominated by terms associated with cell proliferation (Fig. 2), reflecting the anti-CD3 associated activation of T cells. To visualize changes in GO term enrichment and coverage (completeness), immune-associated terms were selected among up- and downregulated DEGs for each antibody treatment, focusing on those associated with the immune response and inflammation typically associated with anti-CD3 therapy (Fig. 3).

All antibodies induced a similar profile of GO term enrichment, coverage and FDR adjusted *p*-value, shown by radar plots (Fig. 3). Among the upregulated genes, the predominance of OKT3-induced GO term coverage was less obvious. Between selected terms, the most enriched GO term among the upregulated genes was the Regulation of Regulatory T Cell Differentiation (GO:0045589), but terms for the regulation of IFNγ (GO:0032729), IL-10 (GO:0032653) and IL-12 production (GO:0032655) were also highlighted.

The downregulated DEG set enriched terms reflected categories that fade after antibody treatment. It is notable that, among the GO terms enriched by genes repressed after treatment, the term “regulation of inflammatory response” (GO:0050727), was the most conspicuous. Furthermore, the terms “immune response-regulating signaling pathway” (GO:0002433) and “activation of immune response” (GO:0002253) were also evident (Fig. 3).

### Regulation of cytokines and their receptors by anti-CD3 stimulation

**Fig. 4:**
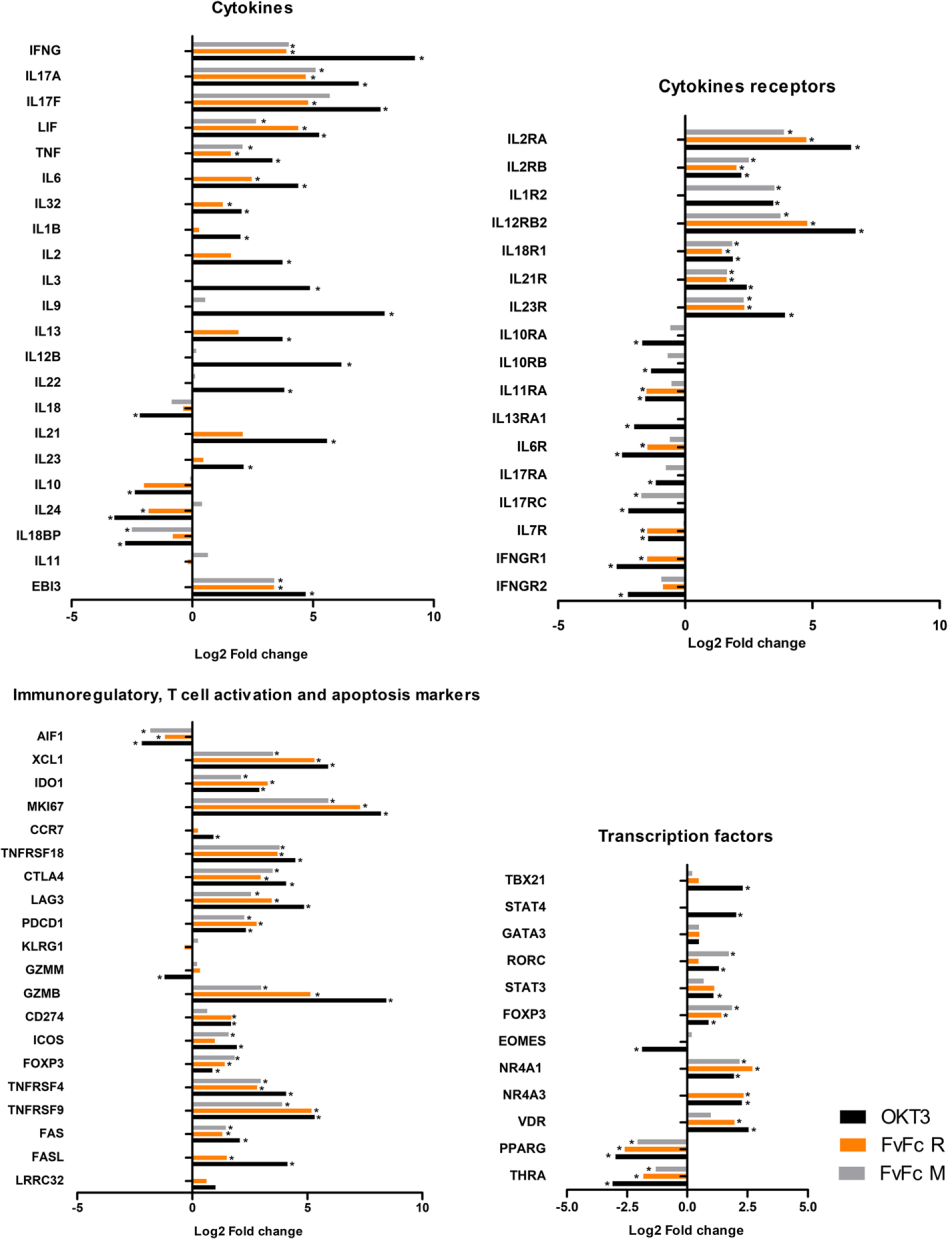
Differentially expressed genes in treated T cells assessed by RNA-seq data. Individual anti-CD3-induced DEG fold changes were grouped according to their biological function. The results are presented as the mean gene expression fold change from two RNA-seq experiments. The asterisk represents padj <0.05. OKT3 (black bars), FvFc R (orange bars) or FvFc M (gray bars)

**Fig. 5:**
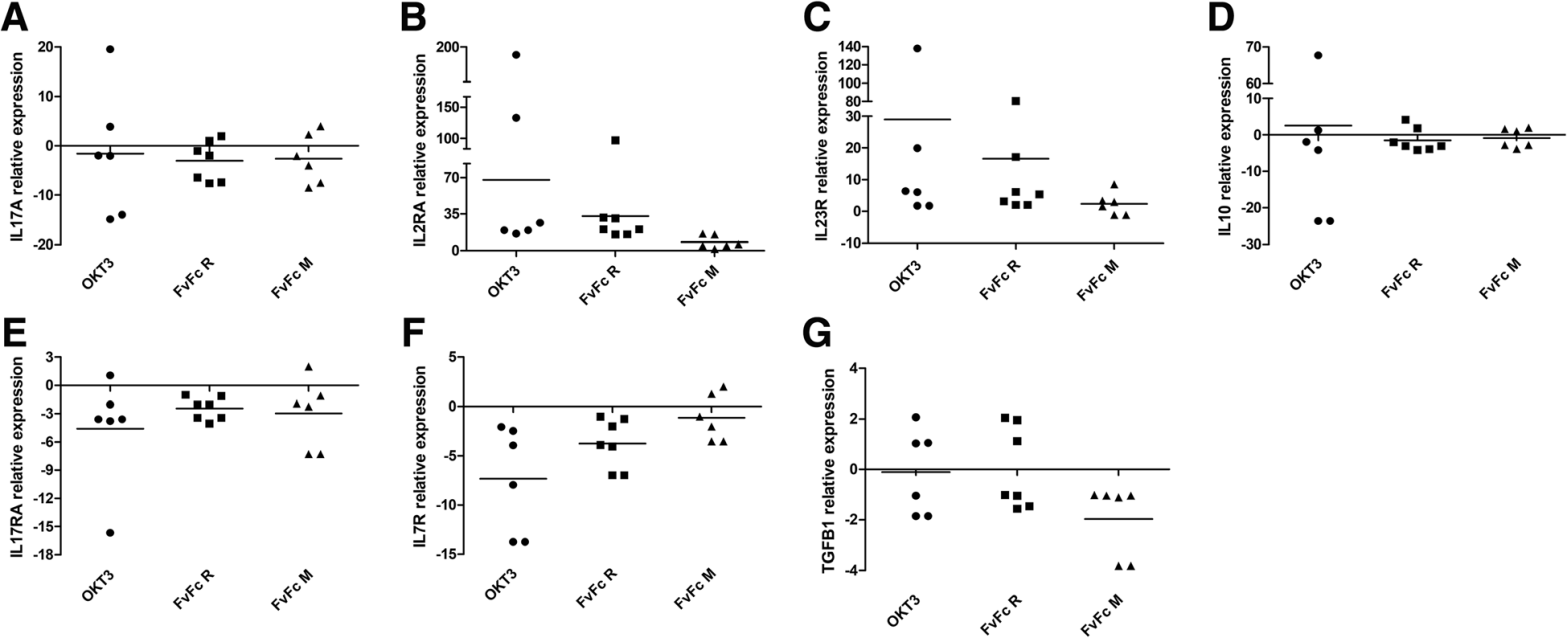
Cytokines and their receptor genes regulated by anti-CD3 stimulation. qPCR assays were performed with total RNA extracted from T cells, 72 h post anti-CD3 stimulation. The results are expressed as the fold change relative to unstimulated T cells (*n* = 7; *p* < 0,05). B2M was used as an internal control for data normalization. **a** IL17A, (**b**) IL2RA, (**c**) IL23R, (**d**) IL10, (**e**) IL17RA, (**f**) IL7R, (**g**) TGFB1

Anti-CD3 antibody therapy is strongly associated with an over secretion of cytokines, also known as a “Cytokine Storm” [4]. The deleterious consequences of the cytokine production are assumed to be promoted by the Fc part of the molecule, and novel humanized antibodies can circumvent these consequences by inducing a nonmitogenic effect. Our data suggest that the in vitro administration of all three anti-CD3 antibodies induce the upregulation of several cytokine genes, including INFG, IL17A, IL17F, LIF and TNF (Fig. 4). However, when we analyzed the expression of IL17 in human donors by RT-qPCR, we noticed that even though the IL17A gene expression was consistently expressed along all treatments in the NGS panel, its induction was variable among antibody-treated donor T cells (Fig. 5a). The FvFc R and OKT3 treatment also induced upregulation of IL6 and IL32. OKT3 treatment induced additional interleukins such as IL1B, IL2, IL3, IL9, IL13, IL12B, IL21 and IL22 (Fig. 4).

Cytokine receptors were also induced after antibody treatment, including strong upregulation of the IL2 receptor subunit genes, IL2RA and IL2RB (Fig. 4). IL2RA expression was also tested in the qPCR panel of treated donor T cells, suggesting that any form of anti-CD3 induces the expression of the IL2 receptor α-chain, also known as CD25 (Fig. 5b). Moreover, all antibody treatments induced the expression of IL1R2, IL12RB2, IL18R1, IL21R, IL23R (Figs. 4 and 5c). However, as suggested by the NGS panel, anti-CD3-treated T cells increased their sensitivity toward IL1, IL2, IL12, IL18, IL21 and IL23.

Anti-CD3 antibody treatment induced the upregulation of several interleukin and interleukin receptors genes, but only a few interleukins and receptors were downregulated due to antibody treatment. IL10 and IL24 expression was significantly repressed after OKT3 and FvFc R treatment, while IL18BP was repressed by OKT3 and FvFc M. In addition, OKT3 treatment also reduced the expression of IL18 (Fig. 4). IL10 was further investigated by qPCR. Notwithstanding, the qPCR panel suggested that OKT3 treatment had a variable effect on IL10 expression among treated donor cells, and the FvFc-based antibody had no significant effect (Fig. 5d).

Downregulation of interleukin receptors makes T cells less sensitive to their cognate cytokine. The NGS panel suggested that OKT3 treatment might interfere with signaling of interleukins IL10, IL11 and IL13, due to the downregulation of IL10RA, IL10RB, IL11RA and IL13RA1 (Fig. 4). IL6R was downregulated after treatment with OKT3 and FvFc R. The IL17RA codes for IL17A specific receptor and was found to be downregulated after OKT3 treatment, with a barely significant q-value (0.0069); nevertheless, the qPCR panel confirmed this tendency for downregulation after treatment with any of the antibodies (Fig. 5e). The IL17RC gene, which codes for a receptor for both IL17A and IL17F, was found to be downregulated after both OKT3 and FvFc M treatment. The receptor for IL7, IL7R, was shown to be downregulated with both FvFc R and OKT3 treatment. The qPCR panel corroborated these results, suggesting that most donor T cells respond to any anti-CD3 antibody format, reducing the IL7R expression levels (Fig. 5f).

### Anti-CD3 stimulation regulates phenotypic marker genes

**Fig. 6:**
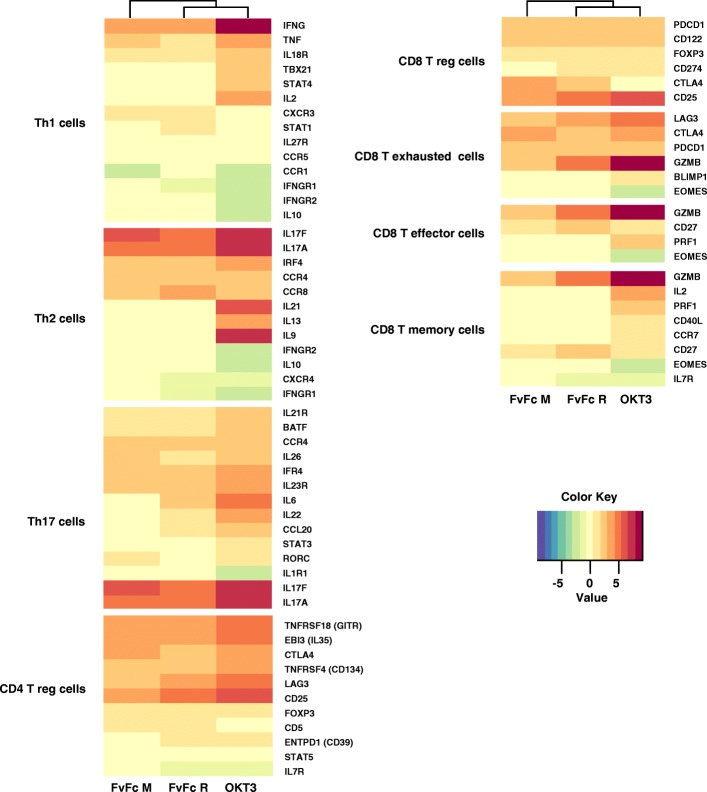
T cell subpopulation signatures. Cluster analysis based on the fold change data of regulated genes (shown in rows) for each sample (columns) and grouped into T cell populations. Only genes with padj ≤ 0.05 were considered differentially expressed. Gradient colors from blue to dark brown represent lower to higher expression (range from − 2.69 to 9.22)

**Fig. 7:**
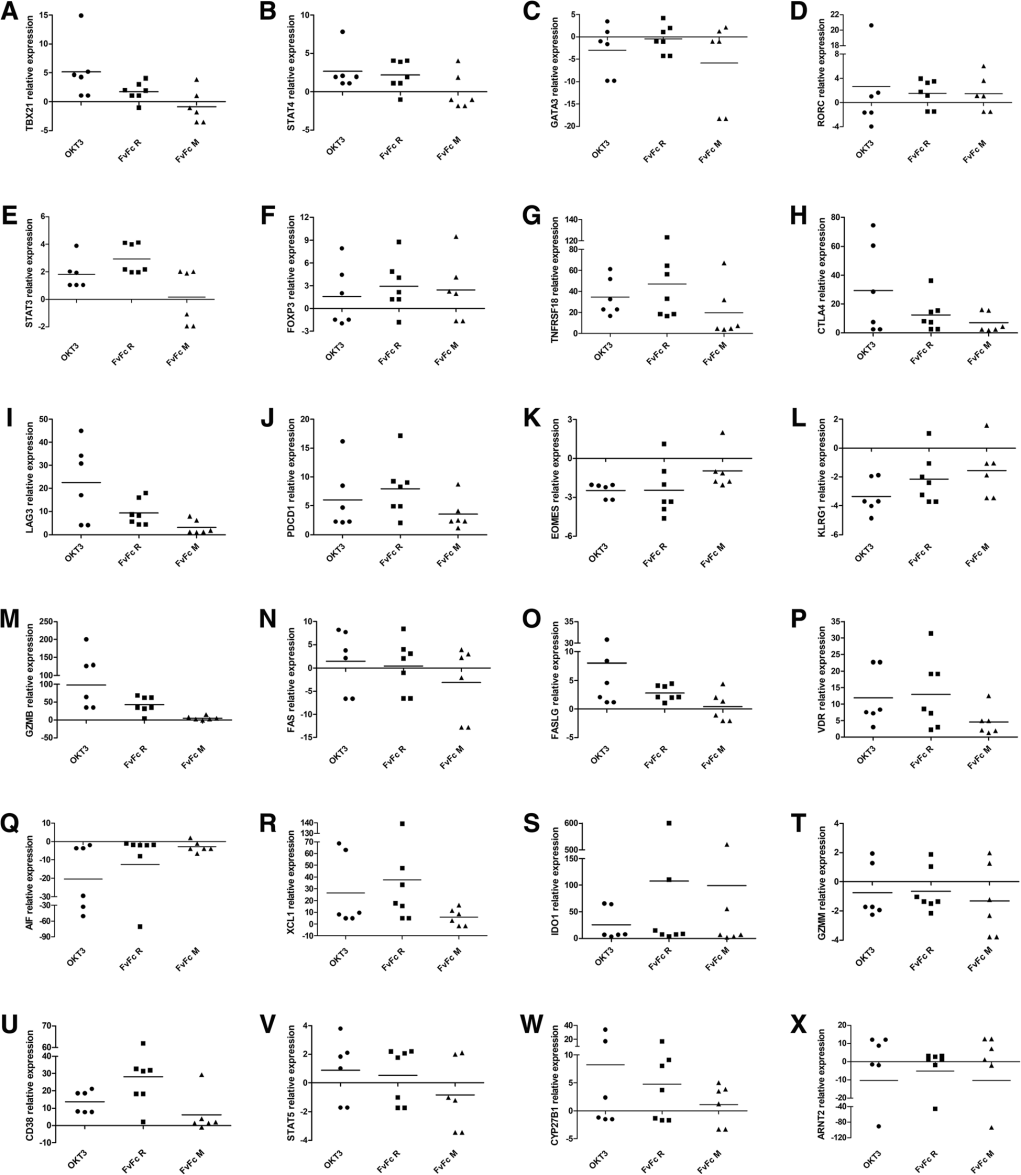
Quantitative analysis of T cell marker expression in anti-CD3-treated T cells. qPCR assays were performed with total RNA extracted from T cells, 72 h post anti-CD3 stimulation; the results are expressed as fold changes relative to levels in unstimulated T cells (*n* = 7; *p* < 0,05). B2M was used as an internal control for data normalization. (**a**) TBX21, (**b**) STAT4, (**c**) GATA, (**d**) RORC, (**e**) STAT3, (**f**) FOXP3, (**g**) TNFRSF18, (**h**) CTLA4, (**i**) LAG3, (**j**) PDCD1, (**k**) EOMES, (**l**) KLRG1, (**m**) GZMB, (**n**) FAS, (**o**) FASLG, (**p**) VDR, (**q**) AIF1, (**r**) XCL1, (**s**) IDO1, (**t**) CD38, (**u**) GZMM, (**v**) STAT5A, (**w**) CYP27B1, (**x**) ARNT2

Activation of resting T cells by anti-CD3 antibodies can induce cell differentiation, and indeed, several phenotypic markers are modulated after antibody treatment. Resting T cells can differentiate in several lineages of effector and regulatory phenotypes, and specific genetic markers can characterize these T cell phenotypes. We compared several markers for CD4 and CD8 subpopulations depicted as panels to visualize their possible differentiation (Fig. 6). To confirm prototype marker expression levels found in the NGS panel, qPCR analyses were performed using anti-CD3 treated T cells (Fig. 7). Some expression markers are key for charactering T cell subpopulations. The Th1 marker TBX21, which codes for the TBET transcription factor, was shown to be significantly induced only with OKT3 treatment in the NGS panel (Fig. 4). The qPCR panel corroborated the NGS data (Fig. 7a), suggesting a minimal effect of FvFc antibodies on TBX21 expression. STAT4, another Th1 marker, was also only induced by OKT3 in the NGS experiment, but qPCR data suggests that FvFc R could also affect the expression levels of STAT4 in stimulated cells [20, 21] (Figs. 4 and 7b). GATA3, a Th2 phenotypic marker, was not significantly induced in NGS or qPCR data (Figs. 4 and 7c). However, other characteristic markers of this subtype were induced [21, 22] (Fig. 6).

In addition, we also analyzed markers for the Th17 subpopulation [23, 24] (Figs. 4, 5, 6 and 7). The gene that codes for ROR_ϒ_t, RORC was found to be slightly upregulated after treatment with both OKT3 and FvFc M antibodies (Fig. 4), but without significance (padj > 0.01). In the qPCR panel, RORC was shown to be barely activated in all three treatments (Fig. 7d). IL17A, known to be produced by Th17 cells, was upregulated in the NGS panel, but these data were not supported by qPCR, which suggests a variable and mild regulation of this gene (Fig. 5a). The third marker, STAT3, was found to be induced by OKT3 in the NGS data and was induced by OKT3 and FvFc R treatments, as measured by qPCR (Fig. 7e). Interestingly, the FvFc M antibody induced a very contrasting effect on different donors. Half of the donors showed an upregulated profile, while the other half showed a downregulated profile.

T cells can assume a regulatory phenotype, and many regulatory markers were found in this analysis [25, 26] (Fig. 6). FOXP3, a major transcription factor that is associated with the human T regulatory phenotype, was upregulated in the NGS data for all antibody treatments. These data were corroborated by qPCR (Fig. 7f). GITR (TNFRSF18) was strongly upregulated by all antibodies (approximately 16-fold, Fig. 4), and this effect was also observed for all donors in qPCR (Fig. 7g). CTLA4 and LAG3 were similarly upregulated in the NGS (Fig. 4), and qPCR data supported this finding (Fig. 7h and i), but the effect was less pronounced for FvFc antibodies compared to OKT3. The gene that codes for PD-1, namely, PDCD1, was also consistently induced by all antibody treatments (approximately 5-fold, Fig. 4), and qPCR data confirmed this observation (Fig. 7j).

Modulations of CD8 T cell markers were also observed after anti-CD3 treatment, suggesting changes in the CD8 T cell population [27, 28] (Fig. 6). Among these markers, EOMES and KLRG1 were repressed after all the anti-CD3 treatments, but GMZB was strongly induced by anti-CD3. These three markers were also tested by qPCR, which confirmed the tendency of the NGS data (Fig. 7k, l and m). Moreover, markers of regulatory CD8 T cell [29], such as IL2RA, were sharply induced by OKT3 and FvFc R but to a lesser extent by FvFc M. CD274 (PD-L1) was only marginally induced in all treatments (Fig. 6), and FOXP3 showed a variable profile (Fig. 7f). ICOS was weakly induced only by OKT3 and FvFc M (Fig. 4).

Phenotypic markers associated with T cell activation, cell death and apoptosis pathways were also affected by anti-CD3 treatment. Figure 4 resumes the induction/repression of these markers after anti-CD3 treatment. Overall, OKT3 induced most activation markers except EOMES and AIF1, while FvFc-based antibodies had a milder profile. Among the activation molecules, IFGN, GZMB, IL2RA, TNFRSF4 and TNFRSF9 showed remarkable induction. Cell death was the fate of activated cells, and the FAS/FASLG pathway was induced after T cell activation. The anti-CD3 effect on FAS induction was slight (Fig. 6) and variable among donors (Fig. 7n), and FASLG was very consistent among donors with the treatment of FvFc R (Fig. 7o). GITR, along with PDCD1, was consistently induced by all the treatments (Figs. 4, 6, 7g and j).

### Anti-CD3 stimulation modulates genes that encode nuclear receptor transcription factors

Nuclear receptors integrate a family of transcription factors that respond to hormones and hydrophobic molecules that have been associated with the control of the immune response [30]. Thus, the PFAM family for Nuclear Receptor (PF00104), was used to probe antibody-induced DEGs. Anti-CD3 treatment induced the expression of PF00104-associated genes. OKT3 induced 7 genes, while FvFc R induced 3 and FvFc M induced 2 genes. The orphan nuclear receptor gene NR4A1 was activated in all treatments at a padj < 10^− 5^. Three other PF00104 annotated genes were found in two of three treatments: NR4A3, RORC, and VDR (Fig. 4). NR4A3 codes for a mitogen-associated nuclear receptor (http://www.uniprot.org/uniprot/Q92570). RORC is mentioned above as a marker for lymphocyte lineages. VDR codes for the vitamin D3 receptor, and its overexpression was detected in all antibody treatments by qPCR (Fig. 7p).

Among the downregulated DEGs, peroxisome proliferator-activated receptor gamma (PPARG), a gene associated with the development of Tregs, was found to be 4- to 9-fold less expressed than that in the unstimulated T cells (Fig. 4). Moreover, the THRA gene that codes for thyroid hormone receptor alpha was also repressed in all treatments.

### Effect of an exclusive anti-CD3 stimulation

To compare the global gene expression profile under the effect of anti-CD3 antibodies with that of activated T cells, we paralleled our results with those described by Zhao and colleagues (2014), who probed DEGs of immortalized T cells cultured in the presence of anti-CD3 and anti-CD28 antibodies. Their DEG dataset after 72 h treatment was compared with NGS data generated in the present work focusing on DEGs regulated after anti-CD3 treatment without the costimulatory anti-CD28 stimulus. Among the 12 most opposite DEG (Additional file 1: Table S3), three genes were selected for qPCR analysis: AIF1, XCL1 and IDO1 (Fig. 7q, r and s). XCL1 and IDO1 were induced by all of the anti-CD3 treatments, as observed in the qPCR panel, while AIF1 was found to be repressed after anti-CD3 treatment.

## Discussion

Anti-CD3 antibodies are known to induce immunosuppression and have been proposed for several therapies, including those for different autoimmune diseases and acute transplanted organ rejection. For approximately two decades, Muromonab-CD3 (OKT3) therapy was used as an adjuvant for acute episodes of graft rejection, but its use was discontinued due to pronounced side effects [2]. However, despite the prolonged clinical use, the mechanism of action of OKT3 is still uncertain. In this study, human T cells were treated with anti-CD3 antibodies in vitro, within the complexity of the PBMC milieu, in an attempt to simulate the natural ambiance that occurs in the intravenous administration of therapeutic anti-CD3. This in vitro experimental model was used to compare the mouse mAb OKT3 with two recombinant antibody fragments inspired by the mAb: a humanized and a chimeric human IgG1 in the FvFc format (scFv-hinge-CH2-CH3).

Currently, most antibody therapies rely on full-sized mAbs, derived from chimeric, humanized or fully human sequences, but new molecular formats may represent technological and economical alternatives. The FvFc format used here represents a novel solution as a single-chained homodimeric molecule that mimics heteromultimeric mAbs [31–36]. The DEG profiles induced by each antibody format were very similar as judged from the enrichment analysis, despite the larger DEG set induced by OKT3, especially for the repressed DEG set. The ontology-based classification for up- and downregulated DEGs suggests that all antibody formats induce a very similar profile, marked by a sharp mitotic response (with a low *p*-value), and a higher, even significant, p-value for “Immune”-related GO. It is noteworthy that FvFc compares positively for several terms, such as regulation of “regulatory T cell” and “interleukin-10 production” and “inflammatory response.” Overall, despite the larger set of OKT3 DEGs, FvFc molecules could enrich GO terms at least similarly.

The broader coverage of GO terms of OKT3 DEGs may reflect their greater mitogenic stimulus [31], while the humanized FvFc displayed a skewed DEG profile, yet preserving its function. Further analyses suggest that the chimeric molecule FvFc R reproduces the OKT3 DEG profile more accurately than FvFc M, despite the better binding proprieties of the latter molecule. Therefore, the humanization process seems to have preserved the original OKT3 paratope in the recombinant molecules, suggesting them as alternative CD3 binders for clinical anti-CD3 therapy.

The mitogenic activity of OKT3 and other anti-CD3 antibodies renders them especially investigated for therapeutics [37–40]. The analysis of differentially expressed gene ontology classification suggests that all three anti-CD3 antibodies modulate a distinct number of genes related to cell proliferation and mitosis. This supports a significant impact of anti-CD3 therapy on T cell proliferation as observed for the proliferation marker MKI67 and the T-specific activation marker CD25 (IL2RA). The activation of T cells by OKT3 and other anti-CD3 antibodies is usually associated with the clinical efficacy of this antibody [39]. However, upregulation of activation markers does not correlate with antibody mitogenic activity, since non mitogenic anti-CD3 antibodies may also induce activation markers *in vivo* [40]. Therefore, these data corroborate a previous characterization of the FvFc R antibody, shown to be less mitogenic than OKT3 [31], despite inducing several activation markers, as observed in the present study.

Most models for anti-CD3 therapy rely on CD4 regulatory cells [41–43], but the majority of data supporting it came from mouse models. Recent data on humanized antibodies in clinical trials highlight the role of CD8 cells in tolerance associated with anti-CD3 therapy, suggesting a two-phase model: a short-term depletion of T cells followed by induction of regulatory mechanisms [6]. A burst of cell activation initially induces mitotic mechanisms. Our data suggest that even after 3 days of anti-CD3 stimulation, activated T cell DEGs are still dominated by a mitotic signature, as seen by GO term enrichment, but, along with, barely detected emerging immunoregulatory mechanisms.

Several regulatory phenotypes have been proposed, along with genes usually associated with a regulatory function [44]. For CD4 cells, regulatory cells are distinguished from effector cells that are classified as Th1, Th2, and Th17. A TBET signature with high production of IFNγ characterizes Th1 cells, but TBX21, which codes for TBET, is only weakly upregulated by OKT3, in line with previous observations [43]. Th2 cells do not appear to be induced by anti-CD3 since no significant alteration in GATA3 expression was observed. Beyond that, markers for Th17 and T regulatory cells are predominantly found in anti-CD3-treated cells. Among those with the Th17 phenotype, IL17A, IL17F, and IL16 were upregulated, and FOXP3, GITR, LAG3, and CTLA4 were characteristic of the regulatory phenotype. These markers were all observed to some degree in each of treatments but commonly were weakly expressed among donors stimulated with FvFc M.

The anti-CD3 treatment seemed to bias toward a Th17/Treg polarity, as suggested before [45, 46]. However, FOXP3, an important marker of regulatory cells, was only weakly induced after PBMC stimulation. It is possible that by analyzing gene expression after 72 h of anti-CD3 induction, we missed the transient FOXP3 peak kinetics [18, 19]. Moreover, the activation of IRF4, a late effect of FOXP3 activation, represses FOXP3 and may negatively affect its expression [47]. IRF4 was upregulated in all the anti-CD3-treated cells. Interestingly, anti-CD3-treated cells showed an apparent decrease in the mRNA levels of CD127 (IL7R), the IL-7 receptor, for which downregulation is considered to be a hallmark of a bona fide regulatory phenotype in humans [29, 48, 49].

IL10 is a marker for regulatory CD8 and the CD4 (Tr1) phenotype [50, 51]. We found no significant IL10 regulation except for a slight decline due to treatment with OKT3. However, we noted an enrichment of the “regulation of Interleukin-10” GO term, suggesting that the machinery for IL10 production was activated in anti-CD3-treated cells. FvFc R antibody was previously shown to induce a high IL10/IFNγ ratio compared to OKT3 in anti-CD3-stimulated PBMCs [31], although no significant induction of IL10 was observed in the present study. Nevertheless, the increase in the IL10/IFNγ ratio observed by Silva and colleagues could be explained in part by a more consistent induction of the IFNG gene in OKT3-treated T cells, or likewise due to a non-lymphocytic origin of the produced IL10 probed in the whole PBMCs [31].

Clinical data on novel humanized antibodies suggest new mechanisms of anti-CD3 action in humans. In mice, studies have suggested that anti-CD3 therapy induces immunosuppression dependent on CD4 T cells, with stimulated helper cells developing a regulatory phenotype. However, in humans, the CD8 lineage also seems to contribute to the tolerogenic effect of anti-CD3, either by inducing differentiation into CD8 regulatory cells [15, 29] or by leading CD8 T cells to exhaustion [11, 12]. Data from Teplizumab clinical trials suggested that the immunosuppressive effect of the humanized antibody is due to anergic and exhausted CD8 cells [12, 52], along with CD8 and CD4 Tregs [15]. Nonetheless, inhibitory receptors were clearly activated in our model system, including PDCD1, CTLA4, and LAG3, suggesting that inhibition of the immune response and inflammation after 72 h of a proliferative stimulus might have led to an exhausted phenotype [14]. Otherwise, inhibitory receptors may not indicate exhaustion, but a detuning of CD8 T activation. It is possible that these inhibitory receptors signify the transition from a highly activated T cell state toward a differentiation/memory profile [11, 53]. The high and consistent induction of the PDCD1 gene (PD-1) observed here suggests a general detuning of anti-CD3-treated cells, that may underlie the basis of anti-CD3 therapeutic effects.

The detuning of T cell following PD-1 expression may contribute to the effect of anti-CD3, but other molecular players may also contribute to immunosuppression. IDO1 is a tryptophan catabolic enzyme known to induce regulatory T cells and immunosuppression [54, 55]. Although usually produced by monocytes, a CD4 + IDO+ lymphocyte population had been characterized [56]. Anti-CD3 treatment induced IDO1 upregulation in T cells, although not uniformly among donors. In this sense, a putative IDO-producing T cell could trigger a profound regulatory effect by locally restricting available tryptophan. This finding may represent an alternative mechanism of T-cell-induced immunosuppression that could be therapeutically exploited.

## Conclusions

Novel therapeutic anti-CD3 antibodies development could focus on regulatory associated GO term enrichment and specific subpopulation markers. The in vitro assay proposed here, based on a simple and economical procedure, seems to be efficient to compare novel antibody molecules before clinical evaluation. Development of new antibodies or novel pharmaceutical association could benefit from this in vitro methodology, allowing a novel discovery pipeline based on a System Biology approach.

In conclusion, we used a deep transcriptome sequencing method for comparing three anti-CD3 antibodies regarding Gene Ontology enrichment and immunological marker expression. The present data showed that both recombinant antibodies induced a compatible expression profile, suggesting that they might be candidates for closer evaluation concerning their therapeutic value. Moreover, the proposed methodology is amenable to be more generally applied for molecular comparison purposes.

## Methods

### Donors

Peripheral blood was collected from seven healthy individuals enrolled in this study (Additional file 1: Table S1). For NGS a single donor was analyzed and for qPCR assays, seven healthy individuals were enrolled. All human blood experiments were performed in accordance to the Ethics Committee of the University of Brasilia guidelines, which approved the study protocol (CAAE: 32874614.4.0000.0030). A written informed consent was obtained from all human donors.

### Antibodies

OKT3 was purchased from eBioscience (San Diego, CA, USA). The humanized antibody fragment FvFc R is a single-chain FvFc molecule and was previously described (FvFc version R) [31]. The chimeric FvFc M contains the original OKT3 VH and VL coding sequences fused to human IgG1 Fc and cloned in the pMIRES expression vector. The FvFc antibody fragments were affinity purified from supernatants of CHO-K1 transfected cells.

### PBMC stimulation and T cell preparation

Fresh PBMCs were isolated using Ficoll-Paque density gradient centrifugation (GE Healthcare, Uppsala, Sweden). PBMCs were cultured in RPMI media (Invitrogen, Carlsbad, CA, USA) supplemented with 4 mM L-glutamine and 10% FBS in the presence or absence of soluble anti-CD3 antibodies. A total of 250 ng of antibody was applied to 1 × 10 [6] PBMC/mL. T cells were isolated after 72 h of PBMC treatment, using magnetic beads by negative selection (according to the manufacturer’s instructions). Briefly, PBMCs were selectively depleted of CD16, CD19, CD20 cells and were discarded (Dynabeads® Untouched™ Human T Cells Kit, Invitrogen, Carlsbad, CA, USA). The purity of T cell enrichment was checked using flow cytometry (Additional file 1: Figure S1).

### RNA extraction

Total RNA was extracted from T cells isolated after PBMC stimulation using the miRNeasy® Mini Kit (Qiagen, Valencia, CA, USA) as described before [46]. RNA integrity and purity were evaluated using a Bioanalyzer 2100 (Agilent Technologies Genomics, Santa Clara, CA, USA). All RNA samples used in this work showed an RIN > 7.

### Sample sequencing and differential gene expression analysis

RNA-seq was performed by Macrogen, Inc. (Seoul, Korea), using the Illumina HiSeq™ 2500 platform (Illumina, San Diego, CA, USA) according to the manufacturer’s standard protocol. The total RNA, up to 1500 ng, was sent in a RNA-stable tube (Biomatrica, Inc., San Diego, CA) to preserve the integrity of the RNA, and Illumina sequencing was performed in a 2 × 150 nt paired-end mode. All sequencing reads produced by Illumina were analyzed for quality control using FASTQC [57]. The reads were aligned to the human genome GRCh37/hg19 downloaded from the UCSC Genome Browser [58] using open source Segemehl, version 0.2.0 [59] with the split read option -S. The aligned files were ordered and indexed using Samtools [60] followed by read counts using HTSeq-count [61]. For a differential gene expression analysis, the reads of CD3 T cell treated and untreated samples uniquely aligned by Segemehl were used. To identify differentially expressed genes (DEGs) for each treatment comparison (treated versus untreated), two replicates per condition were analyzed using the Bioconductor package DESeq2 [62] applying a significance threshold for the adjusted *p*-values of 0.05.

### Analysis of gene functions

The enrichment GO terms for biological processes of DEG were also assessed. For this purpose, upregulated (log_2_FC > 1.2) and downregulated (log_2_FC < 1.2) gene set enrichment analyses were performed using functional categories of the database Gene Ontology (GO). The Panther software [63, 64] was used to calculate enrichment, p-values and FDR adjusted p-values. The super category “biological process” was used, and within this category, GO terms related to the immune system and inflammatory process were further investigated.

Nuclear receptor analysis was performed exclusively for the Pfam family PF00104 of the Pfam database (http://pfam.xfam.org/). Members of PF00104 were searched in the DEG set using regular expression and analyzed individually.

### Gene expression analysis by qPCR assays

Quantitative PCR was performed as previous described [46]. Briefly, total RNA isolated from T cells was utilized for cDNA synthesis using an RT^2^ First Strand Kit (Qiagen, Valencia, CA, USA). The expression genes were quantified using RT^2^ qPCR SYBR Green/ROX MasterMix (Qiagen, Valencia, CA, USA) following the manufacturer’s instructions. The housekeeping gene B2M was used as the endogenous control. qPCR assays were performed using an ABI Step One Plus Real-Time PCR System (Applied Biosystems, Austin, Texas, EUA) and the 2^-ΔΔCt^ method was used to calculate mRNA transcript levels (fold change) using RT^2^ Profiler PCR Array Data Analysis software (SABiosciences, Frederick, MD, USA for analysis. Three independent experiments were performed in triplicate.

### Statistical analysis

All RNA-seq experiments statistical evaluations were performed using Bioconductor package DESeq2 [62] based in Benjamini-Hochberg method for adjusted p-values. Gene function attribution was performed using adjusted p-values calculated by the Binomial statistic and Mann-Whitney U Test (Wilcoxon Rank-Sum Test) by Panther software [63, 64]. Real Time qPCR p-values were calculated based on Student’s t-test using RT^2^ Profiler PCR Array Data Analysis software.

## Additional file

Additional file 1: **Table S1** Donors information. **Figure S1** Analysis of the purity of T cell enrichment. After the enrichment of T cells, the sample was incubated with antibody anti-CD18 FITC, anti-CD3 APC and anti-CD4 PE. A) graphical representation of the physical characteristics of the cells determined by dispersion, B) expression of the CD18 molecule, C) expression of the CD3 molecule within the CD18 population. **Figure S2** Recombinant FvFc forms compete with OKT3 antibody for binding to the CD3 surface molecules on human PBMCs. Lymphocytes were gated in a forward versus side scatter dot plot, and the binding of the anti-human CD3 antibodies was plotted as a histogram. The decreased median fluorescence intensity reflects the inhibition of the FITC conjugated OKT3. **Table S2** Inhibition of OKT3 binding to CD3 molecules in human PBMCs by FvFc forms. **Figure S3** Principal component analysis of RNA-seq reads. **Table S3** Data from DEG for 72 h treatment of Jurkat cell with anti-CD3 and anti-CD28 was obtained from Zhao et al. (2014), supplementartay data, and compared with data for each anti-CD3 treated human T cell for the current work. (PDF 759 kb)

## Data Availability

RNA-Seq datasets are available at GEO (http://www.ncbi.nlm.nih.gov/geo) under the accession number GSE112899. Supplementary informations are in Additional file.pdf.

## Abbreviations

DEGs: Differentially Expressed Genes
FvFc: antibody format - scFv fused to human IgG1 Fc
NGS: Next Generation Sequencing
PBMC: Peripheral Blood Mononuclear Cells
qPCR: Quantitative Real Time PCR
