# Supplementary material for: Gene expression profile of human T cells following a single stimulation of peripheral blood mononuclear cells with anti-CD3 antibodies
Source: BMC Genomics. 2019 Jul 19;20:593. doi: 10.1186/s12864-019-5967-8 (PMC6642599; doi:10.1186/s12864-019-5967-8)
Supplement: Supplementary file 1 — Table S1 Donors information. Figure S1 Analysis of the purity of T cell enrichment. After the enrichment of T cells, the sample was incubated with antibody anti-CD18 FITC, anti-CD3 APC and anti-CD4 PE. A) graphical representation of the physical characteristics of the cells determined by dispersion, B) expression of the CD18 molecule, C) expression of the CD3 molecule within the CD18 population. Figure S2 Recombinant FvFc forms compete with OKT3 antibody for binding to the CD3 surface molecules on human PBMCs. Lymphocytes were gated in a forward versus side scatter dot plot, and the binding of the anti-human CD3 antibodies was plotted as a histogram. The decreased median fluorescence intensity reflects the inhibition of the FITC conjugated OKT3. Table S2 Inhibition of OKT3 binding to CD3 molecules in human PBMCs by FvFc forms. Figure S3 Principal component analysis of RNA-seq reads. Table S3 Data from DEG for 72 h treatment of Jurkat cell with anti-CD3 and anti-CD28 was obtained from Zhao et al. (2014), supplementartay data, and compared with data for each anti-CD3 treated human T cell for the current work. (PDF 759 kb) [file 12864_2019_5967_MOESM1_ESM.pdf]

Supplementary Information

Gene expression profile of human T cells following a single stimulation of peripheral blood mononuclear cells with anti-CD3 antibodies

Correspondence: brigido@unb.br

Supplementary Table 1: Donors information.

Donors	Age	Gender
1	25-30	F
2	25-30	F
3	25-30	F
4	25-30	F
5	25-30	M
6	25-30	M
7	20-25	M

Healthy PBMC donors; Age range in years; F, female; M, male.

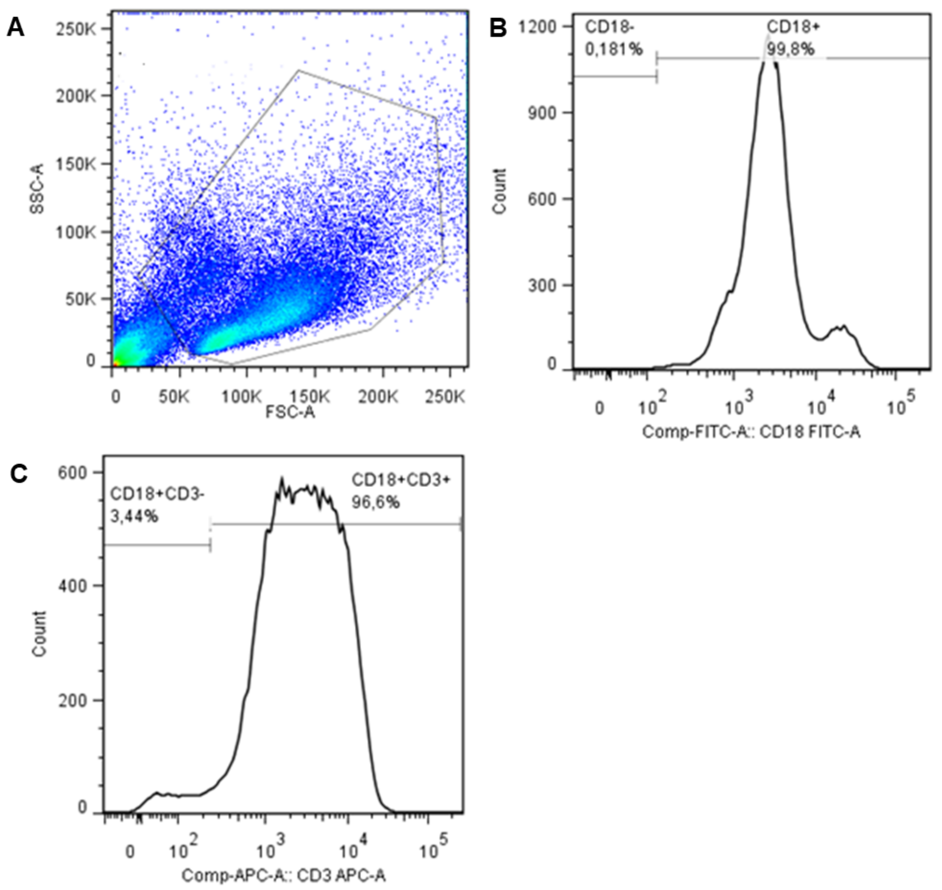

Supplementary Figure 1. Analysis of the purity of T cell enrichment. After the enrichment of T cells, the sample was incubated with antibody anti-CD18 FITC, anti-CD3 APC and anti-CD4 PE. A) graphical representation of the physical characteristics of the cells determined by dispersion, B) expression of the CD18 molecule, C) expression of the CD3 molecule within the CD18 population.

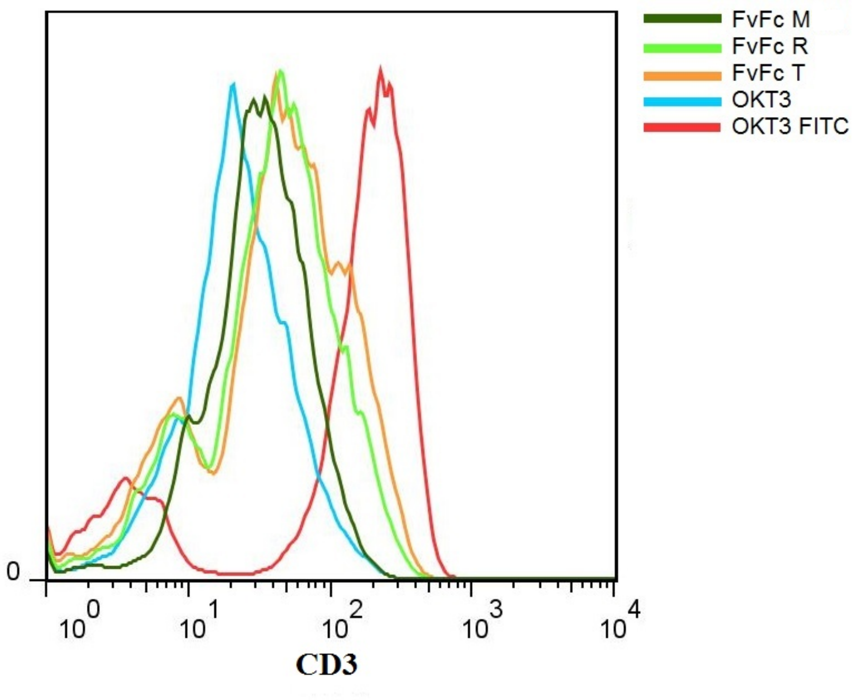

Supplementary Figure 2. Recombinant FvFc forms compete with OKT3 antibody for binding to the CD3 surface molecules on human PBMCs. Lymphocytes were gated in a forward versus side scatter dot plot, and the binding of the anti-human CD3 antibodies was plotted as a histogram. The decreased median fluorescence intensity reflects the inhibition of the FITC conjugated OKT3.

Supplementary Table 2: Inhibition of OKT3 binding to CD3 molecules in human PBMCs by FvFc forms.

Anti-human CD3	FITC median fluorescence intensity	% of FITC conjugated OKT3 binding inhibition	Antibody added (ng)
FvFc T	45.8	84%	312.5
FvFc R	41.8	87%	312.5
FvFc M	31.7	93%	312.5
Unconjugated OKT3	21.6	100%	312.5
FITC conjugated OKT3	173	-	62.5

Human PBMCs were incubated with FITC-conjugated OKT3, washed, incubated with the FvFc forms or unconjugated OKT3 and analyzed by flow cytometry. The percentage of FITC conjugated OKT3 binding inhibition reflects the decrease in FITC median fluorescence intensity when cells were exposed to anti-human CD3 antibodies.

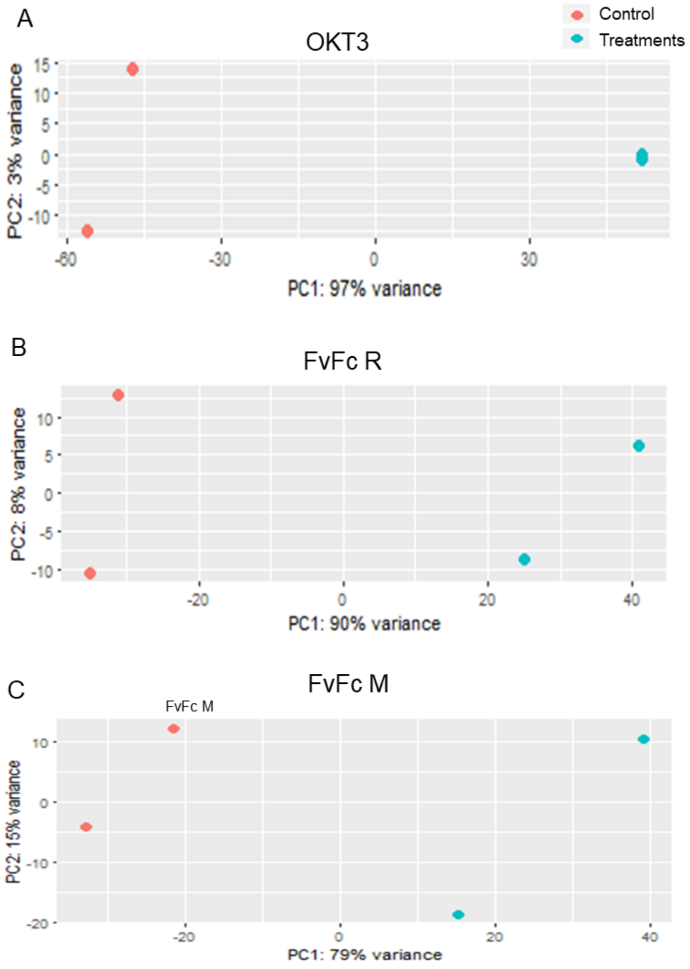

Supplementary Figure 3. Principal component analysis of RNA-seq reads.

Supplementary Table 3: Data from DEG for 72 h treatment of Jurkat cell with anti-CD3 and anti-CD28 was obtained from Zhao et al. (2014), supplementartay data, and compared with data for each anti-CD3 treated human T cell for the current work.

Gene	Zhao* 72h	FcFvR	FvFcM	OKT3
CCL24	(5.989)	2.615	1.687	4.081
RARRES1	0.000	(2.611)	(1.856)	(8.634)
VCAM1	(3.929)	3.358	2.482	4.001
IDO1	(4.840)	2.508	1.508	2.589
ARNT2	(3.626)	2.489	2.497	3.279
XCL1	(0.981)	4.981	3.205	5.669
TNFSF4	(0.585)	3.148	1.772	4.738
AIF1	2.748	(1.130)	(1.672)	(2.111)
CD68	1.072	(1.479)	(2.260)	(3.647)
GBP4	(1.905)	2.651	2.505	2.673
GBP6	(0.808)	1.730	2.335	3.604
GBP5	(1.707)	2.489	2.474	2.524
